# Offering soybean molasses adsorbed to agricultural by‐products improved lactation performance through modulating plasma metabolic enzyme pool of lactating cows

**DOI:** 10.1002/fsn3.2504

**Published:** 2021-10-13

**Authors:** Liang Chen, Hui Mi, Bin Li, Yong Liu, Chuanshe Zhou, Ao Ren, Zhiliang Tan, Zhiwei Kong, Rejun Fang, Ge Zhang

**Affiliations:** ^1^ Key Laboratory for Agro‐Ecological Processes in Subtropical Region National Engineering Laboratory for Pollution Control and Waste Utilization in Livestock and Poultry Production, and Hunan Provincial Key Laboratory of Animal Nutritional Physiology and Metabolic Process Institute of Subtropical Agriculture The Chinese Academy of Sciences Changsha China; ^2^ College of Animal Science and Technology Hunan Agricultural University Changsha China; ^3^ Institute of Animal Science of Tibet Academy of Agricultural and Animal Husbandry Sciences Lhasa China; ^4^ Feng Yi (Shanghai) Biotechnology R&D Center co. LTD Shanghai China

**Keywords:** blood metabolites, lactating dairy cow, milk production, molasses, ruminal simulation

## Abstract

**Background:**

Agricultural by‐products, such as corncob powder (CRP), wheat bran (WB), rice husk (RH), defatted bran (DB), and soybean hulls (SH), were widely used as ruminant feed. However, the combination effect of soybean molasses mixed with agricultural by‐products on cow lactating performance remains poorly understood.

**Methods:**

*In vitro* fermentation simulation technique was used to select the high ruminal fermentation performance of agricultural by‐products mixed with soybean molasses. The selected mixtures were conducted to further explore the feeding effect on milk performance and blood metabolic enzyme on lactating dairy cows.

**Results:**

In *in vitro* simulation, it was confirmed that SH‐SM showed better fermentation performance (including higher maximum gas production, acetate, propionate, and total VFA, but less initial fractional rate of degradation) than other four molasses‐adsorbents, while WB‐SM had the greatest DM and NDF disappearance and NH3‐N and butyrate concentrations among substrates. After the simulation selection, we performed the feed experiment with SH‐SM and WB‐SM compared to the control. For lactating performance, higher (*p* < .01) milk fat and total milk solid content were observed in WB‐SM, and a tendency improvement of milk protein content (*p* < .01) was observed in both of the cows fed with WB‐SM and SH‐SM. Among lactating periods, the blood glutamic‐pyruvic transaminase, α‐amylase, and lactate dehydrogenase which associated with amino acid metabolism and carbohydrate metabolism were improved in lactating dairy cows fed with WB‐SM and SH‐SM.

**Conclusion:**

Dietary agricultural by‐products (like wheat bran and soybean hulls) mixed with soybean molasses enhance the lactating performance of dairy cows by improving the host metabolism process of amino acids and carbohydrates. The mixed strategy for agricultural by‐products shows another strong evidence for the resource reuse on dairy industry and reducing the by‐product pollution.

## Graphical abstract

Dietary agricultural by‐products (like wheat bran and soybean hulls) mixed with soybean molasses enhances the lactating performance of dairy cows by improving the host metabolism process of amino acids and carbohydrates. The mixed strategy for agricultural by‐products shows another strong evidence for the resource reuse on dairy industry and reducing the by‐product pollution.

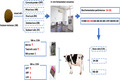

## 1 INTRODUCTION

It is well known that sugars can increase the ruminal fermentability of diet and stimulate dry matter intake (DMI) for ruminants (Firkins et al., 2008; Oelker et al., 2009). It was reported that feeding a sugar‐based product changed the ruminal fermentation pattern, with decreased concentrations of ammonia (NH_3_) (Broderick et al., 2004, 2008) and total volatile fatty acid (TVFA) (Martel et al., 2011) but increased concentrations of ruminal butyrate (DeFrain et al., 2006; Hristov & Ropp, 2003) and milk fat in dairy cows (Martel et al., 2011). This is because most of the sugars could be rapidly fermented in the rumen by the microbes as energy sources, leading to efficient utilization of the rapidly degradable nitrogen fraction and greater microbial protein synthesis, and finally to increase milk protein production (Loman et al., 2016).

Molasses, a sugar‐containing liquid feed, serves as a fat carrier and is also used to enhance mixing of ingredients to prevent feed sorting (Murphy et al., 1997). Previous studies mainly focused on molasses extracted from sugarcane and beet, and supplementation of molasses to dairy cow diets had shown positive effects on lactation performance (Baurhoo et al., 2014; Brito et al., 2015; Cohen‐Zinder et al., 2016; Ghedini et al., 2018). Soybean molasses, a by‐product of soybean meal concentrate, is gaining more researchers’ attention for its high concentration in oligosaccharides, saponins, isoflavones, and other phytochemicals (Shi et al., 2013). Furthermore, supplementation of blended molasses (50% beet sugar molasses and 50% yeast molasses) not only alleviated the decrease of feed intake, but also increased milk production and milk protein content of dairy cows suffering heat stress (Zhang et al., 2013). Broderick and Radloff (2004) reported that replacing high‐moisture corn by molasses improved fiber digestibility, which was likely due to stimulated growth of fiber‐degrading ruminal bacterium by molasses.

Molasses, either offered in dry or liquid form, is a practical source of dietary sugar for feeding to dairy cows (Hall, 2002). Especially, liquid molasses can be used as an alternative delivery vehicle when supplementing mineral elements, rumen‐fermentable carbohydrates, or other phytochemicals in diets of lactating dairy cows (Brito et al., 2017; Shaver et al., 2001). However, current research conclusions are controversial that whether liquid or solid molasses is more beneficial to promote the improvement of lactation performance of dairy cows (Brito et al., 2017; Shaver et al., 2001). Moreover, it was inconvenient to handling storage or transportation of liquid molasses.

Corncob powder (CRP), wheat bran (WB), rice husk (RH), defatted bran (DB), and soybean hulls (SH) are main feed raw materials, characterized with low palatability and energy concentration. Therefore, it was hypothesized that adsorbing these materials with liquid soybean molasses might not only be a good approach to optimize the handling storage and transportation of liquid molasses, but also could possibly enhancing nutritive value of these raw materials. The objectives of the present study were firstly to evaluate the *in vitro* fermentation characteristics of five different feeds that adsorbed soybean molasses using batch culture and secondly two molasses‐adsorbed substrates were chosen to investigate their effects on the responses of milk production performance and blood biochemical parameters of lactating dairy cows.

## 2 MATERIALS AND METHODS

The experiments were conducted according to the animal care guidelines of the Animal Care Committee, Institute of Subtropical Agriculture, The Chinese Academy of Sciences, Changsha City, Hunan Province, China (No. KYNEAAM‐2006–0015).

### 2.1 Experimental Diets and Design

With the objective to explore high utilization of agricultural by‐products, we conducted the lactating dairy cow experiment fed with the agricultural by‐products (ABP) mixed with soybean molasses to improve the lactating performance. First, we used *in vitro* ruminal fermentation simulation technique to select high performance by‐products mixed with soybean molasses. Then with the best 2 selected mixture (by‐product mixed soybean molasses) compared to control, we constructed the complete rations with the mixture to achieve the similar dairy nutrient compositions. The milk performance and blood metabolic enzyme on lactating dairy cows were measured.

### 2.2 *In vitro* Ruminal Simulation Selection

Five agricultural by‐products, including corncob powder (CRP), wheat bran (WB), rice husk (RH), defatted bran (DB), and soybean hulls (SH), were mixed with soybean molasses at a ratio of 10:3 (DM basis), represented as CRP‐SM, WB‐SM, RH‐SM, DB‐SM, and SH‐SM, respectively. The mixture was dried at 65ºC for 24 hr, ground through a 1‐mm sieve, and stored in a nylon bag until further assay. The chemical compositions of five mixtures (by‐products‐molasses) are listed in Table 1.

**TABLE 1:** Chemical compositions of five soybean molasses adsorbed substrates

Item	Substrates				
	CRP‐SM	WB‐SM	RH‐SM	DB‐SM	SH‐SM
DM (g·kg^−1^)	888.2	912.7	926.1	889.9	883.8
N (g·kg^−1^)	12.5	31.7	13.4	30.4	21.2
CP (g·kg^−1^)	78.13	198.13	83.75	190.0	132.5
TE (Mcal·g)	17.91	18.17	16.70	16.64	17.34
NDF (g·kg^−1^)	564.7	298.7	546.1	236.8	528.5
ADF (g·kg^−1^)	353.0	99.4	454.4	94.4	395.9

Abbreviations: ADF, acid detergent fiber; CRP‐SM, corncob powder as soybean molasses adsorbent; DB‐SM, defatted bran as soybean molasses adsorbent; DM, dry matter; N, nitrogen; NDF, neutral detergent fiber; RH‐SM, rice husk as soybean molasses adsorbent; SH‐SM, soybean hulls as soybean molasses adsorbent; TE, total energy; WB‐SM, wheat bran as soybean molasses adsorbent.

#### 2.2.1 *In Vitro* Gas Production and Sampling

The *in vitro* ruminal fermentation simulation technique to select high performance by‐products mixed with soybean molasses. *In vitro* batch culture artificial saliva was prepared using microelement solution, buffer and reducing agent as described by Tang et al. (2006), and kept anaerobic by continuously pumping carbon dioxide for 2 hr. Rumen fluid was obtained from three rumen‐cannulated Holstein dairy cows fed ad libitum a mixed diet of rice straw and concentrate (60:40, wt/wt). The diets were offered twice daily at 0,500 and 1,600 hr. Rumen contents of each dairy cow were obtained from various locations within the rumen before morning feeding, mixed, and strained through four layers of cheesecloth under a continuous CO_2_ stream. The obtained rumen fluid was then anaerobically combined with *in vitro* batch artificial saliva in a proportion of 1 to 9 at 39ºC.

A 1,000 ± 3 mg sample of each substrate was accurately weighed into a 100‐ml fermentation bottle (Wanhong Glass Instrument Factory, China) prewarmed at 39ºC, and then, 50 ml of the mixed fluids (rumen fluids: artificial saliva = 1:9, v/v) were dispensed into each bottle. Nine bottles were prepared for each substrate, and bottles either containing only mixed fluids or substrates were incubated as blanks. All of the fermentation bottles were connected with pressure sensors (CYG130‐12, SQ sensor, China) and incubated at 39ºC. The gas pressure was recorded at 0, 1, 2, 4, 6, 12, 24, and 48 hr. Three bottles for each substrate were removed from the incubator and ended fermentation on ice at 12, 24, and 48 hr, and the pH of fermenting fluids in each bottle was measured immediately, followed by fermentation fluid sampling for determination of NH_3_‐N and VFA concentrations. Then, the undegraded residues were filtered through 2 layers of nylon cloth (40‐µm pore size), dried, and used for determining *in vitro* DM and NDF disappearances.

### 2.3 Animal, Experimental Design, and Sampling

According to the *in vitro* simulation screening, WB‐SM and SH‐SM were selected for further evaluating their effects on milk performance of dairy cows. Twenty‐four multiparous Holstein cows (534 ± 58 kg BW, 2.8 ± 0.7 parity, 60 ± 5 days in milk) were assigned to a randomized complete block design of three groups, with eight individual cows in each group. The experiment treatments were as follows: control (CON), basal diet; SH‐SM, partially replaced corn meal (100 g) and wheat bran (50 g) in the basal diet with 150 g of WB‐SM; and WB‐SM, partially replaced wheat bran (50 g) in the basal diet with 150 g of WB‐SM. The experimental diets (Table 2) were formulated to meet the nutrient requirements of lactating cows according to NRC (2001). The experiment lasted for 90 days, and milk and blood samples were collected for continuous 5 days at the lactating days of 66–70, 96–100, and 126–130, respectively. Cows were housed in a tie‐stall facility throughout the trial, and cows were given a 2‐week period to adapt to the experimental diets. Diets were offered ad libitum twice daily at 0,500 and 1,600 hr, and cows have free access to clean water.

**TABLE 2:** Ingredients and chemical composition of experimental diets

Ingredients	Group		
	CON	SH‐SM	WB‐SM
Feeding (kg/day • cow)			
Rice straw	6.0	6.0	6.0
Beet pulp	3.0	3.0	3.0
DDGS	3.0	3.0	3.0
Concentrate	4.0	4.0	4.0
(g·kg^−1^ of DM)			
Corn meal	431	331	431
Soybean meal	100	100	100
Wheat bran	180	130	30
DDGS	210	210	210
SH adsorbed molasses	—	150	—
WB adsorbed molasses	—	—	150
CaHPO_4_	15	15	15
CaCO_3_	13	13	13
NaHCO_3_	6	6	6
NaCl	5	5	5
Premix^a^	40	40	40
Chemical composition of concentrate (g·kg^−1^of concentrate DM)			
Energy (MJ/kg of DM)	16.7	16.5	17.6
Dry matter	939.8	941.1	946.3
Crude protein	188.9	196.6	191.3
Calcium	19.0	18.6	18.9
Phosphorus	8.0	7.7	7.9
Neutral detergent fiber	187.0	172.2	180.3
RDP, g·kg^−1^ of CP	598.1	601.4	592.9

Abbreviations: DDGS, distillers dried grains with solubles of corn or sorghum; SH‐SM, soybean hulls as soybean molasses adsorbent; WB ‐SM, wheat bran as soybean molasses adsorbent.

^a^Premix (g·kg^−1^): 113.85 g MgSO_4_•H_2_O, 2.69 g FeSO_4_•7H_2_O, 2.55 g CuSO_4_•5H_2_O, 9.54 g MnSO_4_•H_2_O, 9.60 g ZnSO_4_•H_2_O, 0.030 g Na_2_SeO_3_, 0.060 g KI, 0.180 g CoCl_2_•6H_2_O, 500,000 IU Vitamin A, 60 kIU Vitamin D, 2000 IU Vitamin E.

#### 2.3.1 Sampling and Data Collection

The diets offered and left behind were recorded daily for calculating DMI, and feed and residue samples were collected for chemical analysis, as well. Cows were milked twice daily, and individual milk yield was recorded at each milking throughout the trial. Daily milk samples of each dairy cow were collected at both of milking time in the a.m. and p.m. of each sampling period (5 consecutive sampling days) for milk composition analysis. The concentrations of fat, protein, and lactose were determined, and yields of total solids (TS) and solids‐not‐fat (SNF) were calculated corresponding to milk yield. Blood samples were collected from coccygeal vein into vacuum bottles (10 ml) with heparin sodium on the last day of each sampling period at 0,500, 0,700, and 1,100 hr, respectively. The blood samples were kept on ice and immediately transported to the laboratory for centrifugation at 4,000 × *g* for 10 min at 4ºC, and plasma was stored at −80ºC until analysis.

### 2.4 Chemical Analysis

The DM and CP of *in vitro* fermentation substrates, feed, and residue samples were analyzed using the procedures according to AOAC (2016). The NDF and ADF contents were determined using a Fibretherm Fiber Analyzer (Gerhardt, Bonn, Germany) according to Van Soest et al. (1991) with addition of sodium sulfite and α‐amylase in the NDF analysis. The filtered residues were dried at 105 ºC for 2 hr and weighed for determining the *in vitro* DM disappearance (**IVDMD**). The NDF of dried residues was determined to calculate the *in vitro* NDF (**IVNDFD)**. Total gross energy (**TE**) was determined by an isothermal automatic calorimeter (5E‐AC8018, Changsha Kaiyuan Instruments Co., Ltd, China). The NH_3_‐N and VFA concentrations were determined according to Chen et al. (2017). Milk samples were analyzed for fat, protein, lactose, SNF, and TS by mid‐infrared methods (Foss North America, Eden Prairie, MN; Ag‐Source, Verona, WI). Blood chemistry including glutamic‐pyruvic transaminase (GPT), plasma ammonia (AMM), amylase (AMY), cholesterol (CHO), glucose (GLU), lactate dehydrogenase (LDH), triglyceride (TG), total protein (TP), and urea nitrogen (UN) was analyzed by kits (Beijing Leadman Biochemical Co., Ltd, Beijing, China) using auto‐biochemical analyzer (Beckman CX4, Beckman Coulter, Inc. USA).

### 2.5 Data Processing and Statistical Analysis

During the initial stages of the *in vitro* experiment, the correlation between the pressure in fermentation bottles and gas volumes was measured at 39ºC, and the regression equation was then established:

(1) $$y=1.506x\;(n=20,\;R^{2}=0.999,\;P<0.0001)$$

where *y* represents gas volume (mL) and *x* is the pressure in the bottle (kPa). The measured pressure was then converted to gas production (mL). *In vitro* gas production (GP) at 0, 1, 2, 4, 6, 12, 24, and 48 hr was fitted to a logistic‐exponential equation (Wang et al., 2011):

(2) $$GP=V_{f}*\left(1‐\exp\left(d‐t*k\right)\right)/\left(1+\exp\left(b‐k*t\right)\right)$$

where *GP* represents gas production at *t* time, *V_f_* is the maximum gas production (ml), *k* represents gas production fraction (/h), and *b* and *d* represent the shape of the gas production curve. The time (*t_0.5_*, h) when half of the maximum gas production was achieved and the initial fractional rate of degradation (*FRD_0_*, /h) were, respectively, calculated by employing the following two equations (Wang et al., 2011, 2013).

(3) $$T_{0.5}=\text{In}\left(\exp\left(b\right)+2\exp\left(d\right)\right)/k$$

(4) $$FRD_{0}=k/\left(1+\exp\left(b\right)\right)$$

The GP, IVDMD, and IVNDFD were corrected by subtracting the values obtained for the blanks. Data were analyzed by two‐way ANOVA using the MIXED procedure of SAS (2001), and the incubation time was considered as a repeated factor. Results of milk production, milk composition, and blood parameters were statistically analyzed using ANOVA and the MIXED procedure of SAS (2001). The sampling period for milk and blood samples was considered as repeated measurements. Duncan's multiple range tests were used to compare differences among the three treatments. A *p*‐value <0.05 indicated statistical significance.

## 3 RESULTS

### 3.1 *In vitro* Experiment

#### 3.1.1 *In Vitro* Gas Production, IVDMD, and IVNDFD of Different Molasses‐Adsorbents

Both maximum gas production (*V_f_*) and *t_0.5_* of SH‐SM were greater (*p* < .01) than that of CRP‐SM, WB‐SM, RH‐SM, and DB‐SM, among which no differences were observed (Table 3). In the contrast, SH‐SM had the lowest (*p* < .01) *FRD_0_* (0.022 ml/hr) among all molasses‐adsorbents. The IVDMD differed greatly (*p* < .01) among substrates, with the greatest for WB‐SM (69.82%), followed by DB‐SM, SH‐SM, and CRP‐SM, and the lowest in RH‐SM. As for the IVNDFD, WB‐SM and SH‐SM had greater (*p* < .01) value than that of other three molasses‐adsorbents, with the lowest IVNDFD observed for RH‐SM (4.32%).

**TABLE 3:** Effects of different substrates adsorbed to soybean molasses on *in vitro* gas production parameters, IVDMD and IVNDFD

Items	Substrates					SEM	*p*
	CRP‐SM	WB‐SM	RH‐SM	DB‐SM	SH‐SM		
*V_f_* (ml)	169.7^B^	189.1^B^	158.5^B^	171.2^B^	323.4^A^	27.01	<.01
*FRD_0_* (ml·h^−1^) (10^−2^)	3.89^B^	6.42^A^	6.59^A^	6.79^A^	2.20^B^	0.790	<.01
*T_0.5_* (h)	16.71^B^	10.95^B^	10.5^B^	10.09^B^	31.37^A^	2.160	<.001
IVDMD (%)	42.50^D^	69.82^A^	35.36^E^	66.81^B^	52.56^C^	3.9	<.01
IVNDFD (%)	17.41^C^	34.42^A^	4.32^D^	24.89^B^	33.71^A^	8.9	<.01

Abbreviations: CRP‐SM, corncob powder as soybean molasses adsorbent; DB‐SM, defat bran as soybean molasses adsorbent; *FRD_0_*, initial fractional rate of degradation; IVDMD, *in vitro* DM disappearance; IVNDFD, *in vitro* NDF disappearance; RH‐SM, rice husk as soybean molasses adsorbent; SEM, standard error of means; SH ‐SM, soybean hulls as soybean molasses adsorbent; *V_f_*, maximum gas production; WB‐SM, wheat bran as soybean molasses adsorbent.

^A–E^ Means simulation fermentation profiles within a row for different soybean molasses adsorbents combined soybean molasses as fermentation substrates that do not have a common superscript differ (*p* < .05).

#### 3.1.2 *In Vitro* Fermentation Characteristics of Incubation Fluids for Different Molasses‐Adsorbents

The pH of the *in vitro* fermentation fluids ranged from 5.89 to 6.75, with significant lower (*p* < .01) pH observed in WB‐SM than that of the other four molasses‐adsorbents (Table 4). Meanwhile, WB‐SM had the greatest (*p* < 0. 01) NH_3_‐N concentration (35.2 mg/dl) as compared to the other four molasses‐adsorbents.

**TABLE 4:** Effects of different substrates adsorbed to soybean molasses on *in vitro* fermentation characteristics

Items	Substrates					SEM	*p*
	CRP‐SM	WB‐SM	RH‐SM	DB‐SM	SH‐SM		
pH	6.34^B^	5.89^D^	6.75^A^	6.01^C^	6.00^C^	0.03	<.01
NH_3_‐N (mg/dl)	5.09^D^	35.18^A^	15.91^B^	12.95^C^	12.68^C^	0.35	<.01
TVFA (mmol/L)	56.65^C^	75.99^A^	61.24^C^	69.88^B^	81.99^A^	2.98	<.01
Acetate (mmol/L)	32.07^C^	37.10^B^	32.98^BC^	35.73^BC^	45.29^A^	1.59	<.01
Propionate (mmol/L)	19.44^B^	25.41^A^	19.83^B^	24.81^A^	27.08^A^	1.45	<.01
Butyrate (mmol/L)	4.14^C^	9.42^A^	6.72^B^	7.65^B^	7.47^B^	0.53	<.01
A:P	1.91	1.64	2.08	1.55	2.01	0.21	>.05

Abbreviations:P, the ratio of acetate to propionate; CRP‐SM, corncob powder‐soybean molasses adsorbent; DB‐SM, defat bran‐soybean molasses adsorbent; RH‐SM, rice husk‐soybean molasses adsorbent; SEM, standard error of means; SH‐SM, soybean hulls‐soybean molasses adsorbent; TVFA, total VFA; WB‐SM, wheat bran‐soybean molasses adsorbent.

^A–D^ Means within a row for different soybean molasses adsorbed fermentation substrates that do not have a common superscript differ (*p* < .05).

The greatest (*p* < .01) production of TVFA was observed in SH‐SM (81.99 mmol/L) and WB‐SM (75.99 mmol/L), while the lowest production of TVFA was observed in CRP‐SM (56.65 mmol/L) and RH‐SM (61.24 mmol/L) (Table 4). The acetate concentration in SH‐SM was greater (*p* < .01) than that in the other four molasses‐adsorbents; the propionate concentration in SH‐SM, WB‐SM, and DB‐SM, which were not different, was greater (*p* < .01) than that in CRP‐SM and RH‐SM, whereas the greatest (*p* < .01) concentration of butyrate was observed in WB‐SM, with no differences among RH‐SM, DB‐SM, and SH‐SM, which were greater (*p* < .01) than CRP‐SM. However, there were no differences (*p* > *.05*) in A:P for all the five molasses‐adsorbents.

### 3.2 *In Vivo* Experiment

#### 3.2.1 Milk Performance

The averaged milk yield was 25.0 and 17.0 kg during early‐ and mid‐lactation, respectively, with no differences (*p* > .05) among treatments for either lactation period (Table 5). Similarly, the concentration of milk lactose and SNF did not differ (*p* > .05) among treatments in either early‐ or mid‐lactation periods. The concentrations of milk fat and TS in cows fed WB‐SM were greater (*p* < .01) than cows fed control and SH‐SM treatments in early lactation, whereas no differences (*p* > .05) were observed among treatments in mid‐lactation. The milk protein concentration in WB‐SM and SH‐SM treatments was greater (*p* < .01) than CON during both early‐ and mid‐lactation periods.

**TABLE 5:** Effect of different substrates adsorbed to soybean molasses on milking performance in different lactating periods of dairy cows

Item	Group			SEM	*p*
	CON	WB‐SM	SH‐SM		
DMI (kg/day)	14.82	15.02	14.93	0.321	>.05
Milk Production (kg/day)					
Early lactation (0‐100 days)	24.67	23.05	27.16	0.226	>.05
Middle lactation (100‐200 days)	17.85	17.61	15.33	0.153	>0.05
Lactose (g·kg^−1^)					
Early lactation (0‐100 days)	46.2	46.5	46.1	0.091	>.05
Middle lactation (100‐200 days)	45.6	46.6	47.0	0.68	>.05
Solids‐not‐Fat (g·kg^−1^)					
Early lactation (0‐100 days)	82.2	84.4	81.8	1.07	>.05
Middle lactation (100‐200 days)	85.2	84.9	86.9	0.83	>.05
Milk Fat (g·kg^−1^)					
Early lactation (0‐100 days)	29.6^B^	32.7^A^	28.4^B^	0.93	<.01
Middle lactation (100‐200 days)	31.3	30.5	30.7	0.64	>.05
Total Solids (g·kg^−1^)					
Early lactation (0‐100 days)	109.5^B^	116.3^A^	110.2^B^	1.78	<.01
Middle lactation (100‐200 days)	116.5	115.0	117.2	1.19	>.05
Milk Protein (g·kg^−1^)					
Early lactation (0‐100 days)	25.1^B^	28.5^A^	27.1^A^	0.54	<.01
Middle lactation (100‐200 days)	29.3^B^	31.0^A^	30.9^A^	0.68	<.01

CON, control, basal diet without supplementation of soybean molasses; WB‐SM, treatments that replaced 150 g·kg^−1^ of corn meal by wheat bran‐soybean molasses adsorbent; SH was the treatments that replaced 100 g·kg^−1^ of what bran and 50 g·kg^−1^ corn meal by wheat bran ‐soybean molasses adsorbent; SEM, standard error of means

^A, B^ Means within a row for different soybean molasses adsorbed substrates that do not have a common superscript differ (*p* < 0.05).

#### 3.2.2 Blood Biochemistry Indexes

Plasma concentration of GPT in CON was less (*p* < .01) than that in WB‐SM and SH‐SM treatments during both early‐ and mid‐lactation periods, while there was no difference (*p* > .05) between WB‐SM and SH‐SM (Table 6). Plasma concentration of AMY was greater (*p* < .01) in WB‐SM than in CON during both lactation periods, with no difference between WB‐SM and SH‐SM or between CON and SH‐SM. Similar pattern was observed for plasma LDH during mid‐lactation period. However, lower (*p* < .01) plasma TP concentration was observed in WB‐SM and SH‐SM than in CON during mid‐lactation period, with no difference among treatments during early‐lactation period. There were no differences (*p* > .05) in plasma AMM, CHO, GLU, TG, and UN concentration among three treatments during both early‐ and mid‐lactation periods.

**TABLE 6:** Effects of different soybean molasses‐adsorbents on plasma metabolites in different lactating periods in dairy cows

Item	Group			SEM	*p*
	CON	WB‐SM	SH‐SM		
GPT (U/L)					
Early Lactation (0–100 days)	14.43^B^	19.00^A^	19.16^A^	0.88	<.01
Middle Lactation (100–200 days)	14.64^B^	19.36^A^	19.00^A^	1.65	<.01
AMY (U/L)					
Early Lactation (0–100 days)	72.85^B^	143.25^A^	112.00^AB^	23.46	<.01
Middle Lactation (100–200 days)	94.64^B^	125.40^A^	109.15^AB^	19.53	<.01
LDH (U/L)					
Early Lactation (0–100 days)	741.67	771.56	774.50	31.00	>.05
Middle Lactation (100–200 days)	702.00^B^	848.54^A^	753.93^AB^	42.61	<.01
TP (g/L)					
Early Lactation (0–100 days)	74.78	76.38	74.88	10.25	>.05
Middle Lactation (100–200 days)	81.08^A^	75.42^B^	74.26^B^	12.11	<.01
AMM (umol/L)					
Early Lactation (0–100 days)	34.59	27.53	26.30	10.21	>.05
Middle Lactation (100–200 days)	64.17	31.73	31.07	13.08	>.05
CHO (mmol/L)					
Early Lactation (0–100 days)	4.21	4.04	4.01	1.22	>.05
Middle Lactation (100–200 days)	3.95	4.36	3.89	1.30	>.05
GLU (mmol/L)					
Early Lactation (0–100 days)	3.49	3.08	3.09	0.88	> 0.05
Middle Lactation (100–200 days)	3.11	3.31	3.70	1.10	> 0.05
TG (mmol/L)					
Early Lactation (0–100 days)	0.12	0.15	0.12	0.09	>.05
Middle Lactation (100–200 days)	0.14	0.17	0.18	0.06	>.05
UN (mmol/L)					
Early Lactation (0–100 days)	5.27	6.91	6.54	1.22	>.05
Middle Lactation (100–200 days)	5.60	6.49	6.12	1.35	>.05

Abbreviations: AMM, plasma ammonia; AMY, amylase; CHO, cholesterol; CON was the control group without supplementation of soybean molasses; GLU, glucose; GPT, glutamic‐pyruvic transaminase; LDH, lactate dehydrogenase; TG, triglycerides; TP, total protein; UN, urea nitrogen; WB –SM was the treatments that replaced 150 g·kg^‐1^ of corn meal by wheat bran‐soybean molasses adsorbent; SH‐SM was the treatments that replaced 100 g·kg^‐1^ of what bran and 50 g·kg^‐1^ corn meal by wheat bran‐soybean molasses adsorbent; SEM, standard error of means.

^A, B^ Means within a row for different soybean molasses adsorbed substrates that do not have a common superscript differ (*p* < .05).

## 4 DISCUSSION

### 4.1 Effects of Different Molasses‐Adsorbents on *In vitro* Fermentation Characteristics

In the present study, the fermentation characteristics were quite different when soybean molasses was adsorbed to different agricultural by‐products. These differences were likely due to differences of the physical and chemical characteristics of those adsorbents. The chemical composition of substrates is the main factor that affects the *in vitro* fermentation, while the physical structure (especially the cell wall structure) affects the microorganism adsorption amount or rate, which may also determine the rate of *in vitro* fermentation. *In vitro* maximum gas production (*V_f_*), IVDMD, and IVNDFD reflect the potential of utilization efficiency of fermentable substrates (Metzler‐Zebeli et al., 2012), while *FRD_0_* and *T_0.5_* indicate the fermentation speed of substrates, with the faster *FRD_0_* is, the shorter *t_0.5_* becomes (Wang et al., 2013). It was reported that the maximum gas production was positively related to contents of organic matter, hemicellulose, and crude protein of substrates (He et al., 2017; Khalili et al., 1993), as well as the activity of ruminal microbials in *in vitro* fermentation system. In the present study, WB‐SM had the highest IVDMD and IVNDFD, suggesting that it have the highest nutritional value that can potentially be used. Meanwhile, SH‐SM had the highest *V_f_* and *T_0.5_* but lower *FRD_0_* among the five substrates, indicating a high potential but slow fermentation speed to be fermented by ruminal microbials. The slow fermentation speed might be good for high‐producing lactating dairy cows to avoid rumen acidosis. Therefore, WB‐SM and SH‐SM were selected for further study to evaluate their effects on milk performance and health status of lactating dairy cows.

It has been shown that dietary molasses supplementation can improve nutrient digestibility in lactating cows, particularly for fiber (Broderick et al., 2004). Broderick et al. (2004) reported that replacing high‐moisture corn with molasses improved fiber digestibility, reflecting a stimulatory effect of molasses on fiber‐digesting ruminal bacteria (Broderick et al., 2004).

Ruminants usually possess highly developed systems to maintain ruminal pH value within a physiological range of about 5.5–7.0 (Krause & Oetzel, 2006). In the present study, the pH of *in vitro* incubation fluids ranged from 5.89 to 6.75 for the five SM adsorbents, suggesting suitable conditions for fermentation, microbial growth, and fiber degradation. The lower pH in WB‐SM was consistent with greater TVFA production and higher *in vitro* disappearance of DM and NDF. Ruminal NH_3_‐N is an important nitrogen source for microbial growth and protein synthesis, and its concentration should not be less than 5 mg/dl to maintain a high growth rate of bacteria (Satter & Slyter, 1974). In our study, the NH_3_‐N concentrations in *in vitro* fermentation fluids for all five molasses‐adsorbents exceeded 5 mg/dl, indicating a sufficient amount of NH_3_‐N for ruminal bacterial growth and microbial protein synthesis during the fermentation processes. Meanwhile, the difference in NH_3_‐N concentration among substrates was due to the different crude protein contents of CRP, WB, RH, DB, and SH, with the highest and lowest NH_3_‐N concentration observed in WB‐SM and CRP‐SM, respectively, which had the highest and lowest contents of CP. This agreed with previous reporting that there was a strong correlation between dietary CP content and NH_3_‐N concentration (Broderick & Clayton, 1997). Moreover, feeding a sugar‐based product within a diet can change ruminal fermentation pattern and lead to changes in ruminal NH_3_‐N concentration (DeFrain et al., 2006).

Till recently, the reports about the effect of molasses on VFA production have been controversial. Some reported that molasses addition reduced the ruminal acetate concentration but increased butyrate and propionate concentration *in vitro* and *in vivo* (DeFrain et al., 2006; Ferraro et al., 2009; Hristov et al., 2003), while others reported that dietary molasses supplementation increased the molar proportions of acetate and butyrate, but decreased the proportions of propionate and TVFA in dairy cows (Martel et al., 2011), whereas there were also reports indicating that dried molasses did not alter the ruminal concentration of total VFA or individual VFA (Broderick et al., 2004). In the present study, the concentration of acetate, propionate, butyrate, and TVFA of *in vitro* incubation fluids was significantly different among the five SM adsorbents, which is linked with the physical and chemical characteristics of these adsorbents as mentioned previously. Moreover, the variations in VFA concentration might be associated with the differences in ruminal OM digestibility of five molasses‐adsorbents (Calsamiglia et al., 2008). In the present study, WB‐SM and SH‐SM had greater TVFA than the rest adsorbents, implying that they can provide more energy for ruminants.

Taken together, the *in vitro* fermentation characteristics, including TVFA production, NH_3_‐N concentration, and pH, were consistent with the *in vitro* disappearance of DM and NDF, which were determined by the physical and chemical characteristics of these adsorbents. Comprehensively considering the *in vitro* fermentation characteristics of the five molasses‐adsorbents, especially *in vitro* disappearance of NDF and VFA concentration, two molasses‐adsorbents (*i.e*., wheat bran‐molasses, WB‐SM; soybean hull‐molasses, SH‐SM) were selected for further *in vivo* experiment.

### 4.2 Effects of WB‐SM and SH‐SM on Milk Performance and Blood Metabolites of Lactating Dairy Cows

In the present study, the milk production, milk lactose, and SNF were not affected when partially replacing corn meal with WB‐SM or with SH‐SM (without replace corn meal) during both early‐ and mid‐lactation periods. Our results agreed with Martel et al. (2011) that dietary molasses supplementation did not affect milk yield when replaced corn with molasses at a rate of 50 g/kg dietary DM. However, Baurhoo and Mustafa (2014) reported that milk yield decreased in lactating cows fed flaxseed meal‐based diets replacing corn meal with liquid molasses. These differences might be resulted from the different molasses sources and dietary compositions.

Yield of milk protein was greatly enhanced in WB‐SM and WB‐SM than cows that received basal diets during both early‐ and mid‐lactation periods, indicating a promising way of improve milk quality by supplementing soybean molasses in diets for dairy cows. The increment of milk protein content during early‐ and mid‐lactation periods is consistent with the previous literature (Broderick et al., 2004). Similar results were reported by Yan et al. (1997) that when the inclusion rate of molasses increased from 156 to 468 g/kg, DM improved milk protein concentration from 31.6 to 33.6 g/kg for mid‐lactation cows. Keady and Murphy (1998) observed that supplementing sucrose (10 g/kg DM) significantly increased milk protein concentration of lactating dairy cows. Furthermore, Murphy (1999) suggested that milk protein yield could be increased when dairy cows were fed rumen‐fermentable energy in the form of molasses in a grass silage‐based diet. The above‐mentioned findings support our results given that milk protein content was increased in WB‐SM and SH‐SM treatments during early‐ and mid‐lactation periods. It was suggested that ruminal microbial protein synthesis could be stimulated and a greater proportion of degradable *N* could be captured by rumen microbes for dairy cows, leading to increase milk protein synthesis. The greater NH_3_‐N concentration in the *in vitro* fermentation fluids was also consistent with the greater milk protein yield.

The milk fat and total solids were improved when partially replacing corn meal with WB‐SM in basal diet during early‐lactation period, while no effect was observed during the mid‐lactation period. An increased milk fat content according to dietary molasses supplementation was reported by Martel et al. (2011), which was in agreement with the current study. However, the increased TS was in contrary to previous reports that there was no effect of molasses supplementation on milk TS content in lactating cows (Baurhoo et al., 2014; Brito et al., 2017). Milk solid production is associated with energy intake (Broderick, 2003). In the current study, yield of milk TS was greatest in WB‐SM, which had higher energy‐dense as compared to the other two diets. Although the DMI was not different among treatments, the higher energy density would probably resulted in higher energy intake, which might explain the greater milk ST in WB‐SM. Besides, a higher milk protein and milk fat production in WB diet in early‐lactation were also one of main reason resulted in the increment of milk total solids.

Blood metabolites are usually used to reflect health status of dairy cows. Enzymes like GPT (or ALT) and LDH are often specifically expressed in liver and heart, and their activities remain stable at low levels in blood under healthy conditions, but will increase abnormally to high levels if organ damage occurred. Although greater plasma GPT concentration was observed in the two molasses‐adsorbents treatments than in control treatment, the concentration was still within the normal range of healthy lactating dairy cows as reported previously (Stojević et al., 2005). Similarly, although Noziere et al. (2014) confirmed that high LDH can indicate inflammation, the plasma LDH was also within the normal range during both early‐ and mid‐lactation periods according to Piccinini et al. (2007). Our results indicate that the dairy cows under the current feeding condition remained healthy. In the present study, greater plasma LDH concentration in WB‐SM treatment than that of control during mid‐lactation period was likely due to lactate consumption by lactate‐utilizing bacteria of WB‐SM treatment increased the need to metabolize lactate.

Blood metabolites can also be used to evaluate the metabolic function status of dairy cows. For example, blood TP concentration can be used as an indicator of the long‐term protein status of dairy cows (Topps & Thompson, 1984), while plasma UN concentration reflected dietary protein intake and N utilization efficiency (Thomas et al., 1988). Dairy cows with high genetic merit require an energy‐dense diet to fulfill their production potential, and thus, cereals rich in starch are prevalent in the diets of high‐producing dairy cows (Noziere et al., 2014). In the present study, greater plasma AMY concentration in WB‐SM treatment during early‐ and mid‐lactation periods was likely because molasses were fermented rapidly in the rumen, which supplied energy to the ruminal microbes leading to a more efficient utilization of starch. Previous studies reported no significant differences in blood TP concentration in calves (Lesmeister & Heinrichs, 2005) or Moghani sheep (Azizi‐Shotorkhoft et al., 2013) receiving different levels of dietary molasses. In the present study, cows received WB‐SM or SH‐SM diets had lower plasma TP than that of cows fed control diet, but was still within the normal range. The lack of significant difference in plasma UN concentration in the present study agreed with the findings of Hatfield et al. (1998), who reported that molasses type had no effect on plasma UN in sheep. All these results indicated that the metabolic function was not affected by soybean molasses.

## 5 CONCLUSION

Two molasses‐adsorbents, soybean molasses adsorbed by wheat bran and soybean hulls, improved maximum gas production, ruminal total volatile fatty acid concentration, and neutral detergent fiber degradation *in vitro*. Further *in vivo* experiment showed that dietary supplementation of wheat bran adsorbed soybean molasses increased milk protein, fat, and total solids contents in lactating dairy cows. Further research is needed to better understand the influence on *in vivo* rumen fermentation characteristics and nutrient digestibility and their potential relationships.

## CONFLICTS OF INTEREST

The authors have declared that no conflicts of interests exist.

## AUTHOR CONTRIBUTIONS

**Liang Chen:** Formal analysis (equal); Methodology (lead); Supervision (equal); Writing‐original draft (lead). **Hui Mi:** Methodology (supporting); Writing‐review & editing (equal). **Bin Li:** Conceptualization (equal); Supervision (equal). **Yong Liu:** Formal analysis (equal); Software (equal). **Chuanshe Zhou:** Project administration (lead); Supervision (equal). **Ao Ren:** Methodology (supporting); Visualization (equal). **Zhiliang Tan:** Investigation (equal); Supervision (equal). **Zhiwei Kong:** Conceptualization (equal); Supervision (equal). **Rejun Fang:** Formal analysis (equal); Methodology (equal); Resources (equal); Supervision (equal). **Ge Zhang:** Methodology (equal); Software (equal).

## ETHICAL APPROVAL

The experiments were conducted according to the animal care guidelines of the Animal Care Committee, Institute of Subtropical Agriculture, The Chinese Academy of Sciences, Changsha City, Hunan Province, China (No. KYNEAAM‐2006–0015).

## Data Availability

The data that support the findings of this study are available from the corresponding author, Chuanshe Zhou, upon reasonable request.

## References

[fsn32504-bib-0001] Al Loman, A. , & Ju, L.‐K. (2016). Soybean carbohydrate as fermentation feedstock for production of biofuels and value‐added chemicals. Process Biochemistry, 51(8), 1046–1057. 10.1016/j.procbio.2016.04.011

[fsn32504-bib-0002] AOAC (Association of Official Analytical Chemists) (2016). Official methods of analysis. Association of Official Analytical Chemists.

[fsn32504-bib-0003] Azizi‐Shotorkhoft, A. , Rezaei, J. , & Fazaeli, H. (2013). The effect of different levels of molasses on the digestibility, rumen parameters and blood metabolites in sheep fed processed broiler litter. Animal Feed Science and Technology, 179(1–4), 69–76. 10.1016/j.anifeedsci.2012.12.001

[fsn32504-bib-0005] Baurhoo, B. , & Mustafa, A. (2014). Effects of molasses supplementation on performance of lactating cows fed high‐alfalfa silage diets. Journal of Dairy Science, 97(2), 1072–1076. 10.3168/jds.2013-6989 24315324

[fsn32504-bib-0006] Brito, A. F. , Petit, H. V. , Pereira, A. B. D. , Soder, K. J. , & Ross, S. (2015). Interactions of corn meal or molasses with a soybean‐sunflower meal mix or flaxseed meal on production, milk fatty acid composition, and nutrient utilization in dairy cows fed grass hay‐based diets. Journal of Dairy Science, 98(1), 443–457. 10.3168/jds.2014-8353 25465544

[fsn32504-bib-0007] Brito, A. F. , Soder, K. J. , Chouinard, P. Y. , Reis, S. F. , Ross, S. , Rubano, M. D. , & Casler, M. D. (2017). Production performance and milk fatty acid profile in grazing dairy cows offered ground corn or liquid molasses as the sole supplemental nonstructural carbohydrate source. Journal of Dairy Science, 100(10), 8146–8160. 10.3168/jds.2017-12618 28780091

[fsn32504-bib-0008] Broderick, G. A. (2003). Effects of varying dietary protein and energy levels on the production of lactating dairy cows. Journal of Dairy Science, 86(4), 1370–1381. 10.3168/jds.S0022-0302(03)73721-7 12741562

[fsn32504-bib-0009] Broderick, G. A. , & Clayton, M. K. (1997). A statistical evaluation of animal and nutritional factors influencing concentrations of milk urea nitrogen. Journal of Dairy Science, 80(11), 2964–2971. 10.3168/jds.S0022-0302(97)76262-3 9406089

[fsn32504-bib-0010] Broderick, G. A. , Luchini, N. D. , Reynal, S. M. , Varga, G. A. , & Ishler, V. A. (2008). Effect on production of replacing dietary starch with sucrose in lactating dairy cows. Journal of Dairy Science, 91(12), 4801–4810. 10.3168/jds.2008-1480 19038955

[fsn32504-bib-0011] Broderick, G. A. , & Radloff, W. J. (2004). Effect of molasses supplementation on the production of lactating dairy cows fed diets based on alfalfa and corn silage. Journal of Dairy Science, 87(9), 2997–3009. 10.3168/jds.S0022-0302(04)73431-1 15375061

[fsn32504-bib-0012] Calsamiglia, S. , Cardozo, P. W. , Ferret, A. , & Bach, A. (2008). Changes in rumen microbial fermentation are due to a combined effect of type of diet and pH. Journal of Animal Science, 86(3), 702–711. 10.2527/jas.2007-0146 18073289

[fsn32504-bib-0013] Chen, L. , Ren, A. , Zhou, C. , & Tan, Z. (2017). Effects of Lactobacillus acidophilus supplementation for improving in vitro rumen fermentation characteristics of cereal straws. Italian Journal of Animal Science, 16(1), 52–60. 10.1080/1828051x.2016.1262753

[fsn32504-bib-0014] Cohen‐Zinder, M. , Leibovich, H. , Vaknin, Y. , Sagi, G. , Shabtay, A. , Ben‐Meir, Y. , Nikbachat, M. , Portnik, Y. , Yishay, M. , & Miron, J. (2016). Effect of feeding lactating cows with ensiled mixture of *Moringa oleifera*, wheat hay and molasses, on digestibility and efficiency of milk production. Animal Feed Science and Technology, 211, 75–83. 10.1016/j.anifeedsci.2015.11.002

[fsn32504-bib-0015] DeFrain, J. M. , Hippen, A. R. , Kalscheur, K. F. , & Schingoethe, D. J. (2006). Feeding lactose to increase ruminal butyrate and the metabolic status of transition dairy cows. Journal of Dairy Science, 89(1), 267–276. 10.3168/jds.S0022-0302(06)72091-4 16357290

[fsn32504-bib-0016] Ferraro, S. M. , Mendoza, G. D. , Miranda, L. A. , & Gutierrez, C. G. (2009). In vitro gas production and ruminal fermentation of glycerol, propylene glycol and molasses. Animal Feed Science and Technology, 154(1–2), 112–118. 10.1016/j.anifeedsci.2009.07.009

[fsn32504-bib-0017] Firkins, J. L. , Oldick, B. S. , Pantoja, J. , Reveneau, C. , Gilligan, L. E. , & Carver, L. (2008). Efficacy of liquid feeds varying in concentration and composition of fat, nonprotein nitrogen, and nonfiber carbohydrates for lactating dairy cows. Journal of Dairy Science, 91(5), 1969–1984. 10.3168/jds.2007-0868 18420628

[fsn32504-bib-0018] Ghedini, C. P. , Moura, D. C. , Santana, R. A. V. , Oliveira, A. S. , & Brito, A. F. (2018). Replacing ground corn with incremental amounts of liquid molasses does not change milk enterolactone but decreases production in dairy cows fed flaxseed meal. Journal of Dairy Science, 101(3), 2096–2109. 10.3168/jds.2017-13689 29274976

[fsn32504-bib-0020] Hall, M. B. (2002). Working with sugars (and molasses) (pp. 146–158). Pages in Proc. 13th Annu. Florida Ruminant Nutrition Symp, Gainesville, FL: Florida Dairy Extension.

[fsn32504-bib-0021] Hatfield, P. G. , Hopkins, J. A. , Ramsey, W. S. , & Gilmore, A. (1998). Effects of level of protein and type of molasses on digests kinetics and blood metabolites in sheep. Small Ruminant Research, 28(2), 161–170. 10.1016/s0921-4488(97)00085-0

[fsn32504-bib-0022] He, Y. , Wang, H. , Xu, J. , & Zhao, R. (2017). Evaluation on feeding value of ramie using in vitro gas production and nylon bag methods. Chinese Journal of Animal Nutrition, 29(2), 690–698.

[fsn32504-bib-0023] Hristov, A. N. , & Ropp, J. K. (2003). Effect of dietary carbohydrate composition and availability on utilization of ruminal ammonia nitrogen for milk protein synthesis in dairy cows. Journal of Dairy Science, 86(7), 2416–2427. 10.3168/jds.S0022-0302(03)73836-3 12906060

[fsn32504-bib-0024] Keady, T. W. J. , & Murphy, J. J. (1998). The effects of ensiling and supplementation with sucrose and fish meal on forage intake and milk production of lactating dairy cows. Animal Science, 66, 9–20. 10.1017/s1357729800008791

[fsn32504-bib-0025] Khalili, H. , Varvikko, T. , & Osuji, P. O. (1993). Supplementation of grass hay with molasses in crossbred (Bostaurus × Bosindicus) non‐lactating cows: Effect of level of molasses on feed intake, digestion, rumen fermentation and rumen digesta pool size. Animal Feed Science and Technology, 41(1), 39–50. 10.1016/0377-8401(93)90093-y

[fsn32504-bib-0026] Krause, K. M. , & Oetzel, G. R. (2006). Understanding and preventing subacute ruminal acidosis in dairy herds: A review. Animal Feed Science and Technology, 126(3–4), 215–236. 10.1016/j.anifeedsci.2005.08.004

[fsn32504-bib-0027] Lesmeister, K. E. , & Heinrichs, A. J. (2005). Effects of adding extra molasses to a texturized calf starter on rumen development, growth characteristics, and blood parameters in neonatal dairy calves. Journal of Dairy Science, 88(1), 411–418. 10.3168/jds.S0022-0302(05)72702-8 15591407

[fsn32504-bib-0028] Martel, C. A. , Titgemeyer, E. C. , Mamedova, L. K. , & Bradford, B. J. (2011). Dietary molasses increases ruminal pH and enhances ruminal biohydrogenation during milk fat depression. Journal of Dairy Science, 94(8), 3995–4004. 10.3168/jds.2011-4178 21787935

[fsn32504-bib-0029] Metzler‐Zebeli, B. U. , Scherr, C. , Sallaku, E. , Drochner, W. , & Zebeli, Q. (2012). Evaluation of associative effects of total mixed ration for dairy cattle using in vitro gas production and different rumen inocula. Journal of the Science of Food and Agriculture, 92(12), 2479–2485. 10.1002/jsfa.5656 22450904

[fsn32504-bib-0030] Murphy, J. J. (1999). The effects of increasing the proportion of molasses in the diet of milking dairy cows on milk production and composition. Animal Feed Science and Technology, 78(3–4), 189–198. 10.1016/s0377-8401(99)00007-3

[fsn32504-bib-0031] Murphy, M. R. , Geijsel, A. W. P. , Hall, E. C. , & Shanks, R. D. (1997). Dietary variety via sweetening and voluntary feed intake of lactating dairy cows. Journal of Dairy Science, 80(5), 894–897. 10.3168/jds.S0022-0302(97)76011-9 9178129

[fsn32504-bib-0032] Noziere, P. , Steinberg, W. , Silberberg, M. , & Morgavi, D. P. (2014). Amylase addition increases starch ruminal digestion in first‐lactation cows fed high and low starch diets. Journal of Dairy Science, 97(4), 2319–2328. 10.3168/jds.2013-7095 24534508

[fsn32504-bib-0033] Oelker, E. R. , Reveneau, C. , & Firkins, J. L. (2009). Interaction of molasses and monensin in alfalfa hay‐ or corn silage‐based diets on rumen fermentation, total tract digestibility, and milk production by Holstein cows. Journal of Dairy Science, 92(1), 270–285. 10.3168/jds.2008-1432 19109286

[fsn32504-bib-0034] Piccinini, R. , Binda, E. , Belotti, M. , Dapra, V. , & Zecconi, A. (2007). Evaluation of milk components during whole lactation in healthy quarters. Journal of Dairy Research, 74(2), 226–232. 10.1017/s0022029906002317 17227595

[fsn32504-bib-0036] SAS Institute (Ed.) (2001). The SAS System for Microsoft Windows, release 8.2. SAS Institute Inc.

[fsn32504-bib-0037] Satter, L. D. , & Slyter, L. L. (1974). Effect of ammonia concentration of rumen microbial protein production in vitro. British Journal of Nutrition, 32(2), 199–208. 10.1079/bjn19740073 4472574

[fsn32504-bib-0038] Shaver, R. D. (2001). Recent applications of liquid feed supplements in rations for lactating dairy. Cows, 17(2), 17–19.

[fsn32504-bib-0039] Shi, Y. , Kong, X. , Zhang, C. , Chen, Y. , & Hua, Y. (2013). Adsorption of soy isoflavones by activated carbon: Kinetics, thermodynamics and influence of soy oligosaccharides. Chemical Engineering Journal, 215, 113–121. 10.1016/j.cej.2012.10.100

[fsn32504-bib-0040] Stojević, Z. , Piršljin, J. , Milinković‐Tur, S. , Zdelar‐Tuk, M. , & Ljubić, B. B. (2005). Activities of AST, ALT and GGT in clinically healthy dairy cows during lactation and in the dry period. Veterinary Archives, 75, 67–73.

[fsn32504-bib-0041] Tang, S. , Tan, Z. , Zhou, C. , Jiang, H. , Jiang, Y. , & Sheng, L. (2006). A comparison of in vitro fermentation characteristics of different botanical fractions of mature maize stover. Journal of Animal and Feed Sciences, 15(3), 505–515. 10.22358/jafs/66920/2006

[fsn32504-bib-0042] Thomas, V. M. , Mcinerney, M. J. , & Kott, R. W. (1988). Influence of body condition and lasalocid during late gestation on blood metabolites, lamb birth weight and colostrum composition and production in finn‐cross ewes. Journal of Animal Science, 66(3), 783. 10.2527/jas1988.663783x 3378934

[fsn32504-bib-0043] Topps, J. H. , & Thompson, J. K. (1984). Blood characteristics and the nutrition of ruminants. Stationery Office Books.

[fsn32504-bib-0044] Van Soest, P. J. , Robertson, J. B. , & Lewis, B. A. (1991). Methods for dietary fiber, neutral detergent fiber, and nonstarch polysaccharides in relation to animal nutrition. Journal of Dairy Science, 74, 3583–3597. 1660498 10.3168/jds.S0022-0302(91)78551-2

[fsn32504-bib-0045] Wang, M. , Sun, X. Z. , Tang, S. X. , Tan, Z. L. , & Pacheco, D. (2013). Deriving fractional rate of degradation of logistic‐exponential (LE) model to evaluate early in vitro fermentation. Animal, 7(6), 920–929. 10.1017/s1751731112002443 23290561

[fsn32504-bib-0046] Wang, M. , Tang, S. X. , & Tan, Z. L. (2011). Modeling in vitro gas production kinetics: Derivation of Logistic‐Exponential (LE) equations and comparison of models. Animal Feed Science and Technology, 165(3–4), 137–150. 10.1016/j.anifeedsci.2010.09.016

[fsn32504-bib-0047] Yan, T. , Roberts, D. J. , & Higginbotham, J. (1997). The effects of feeding high concentrations of molasses and supplementing with nitrogen and unprotected tallow on intake and performance of dairy cows. Animal Science, 64(01), 17–24. 10.1017/S1357729800015514

[fsn32504-bib-0048] Zhang, W. , Li, S. , Shi, H. , Luo, Y. , Yang, J. , Yin, Y. , & Jia, C. (2013). Different kinds of molasses: Effects on feed intake and milk performance of dairy cows during summer heat stress. Chinese Journal of Animal Nutrition, 25(1), 163–170.

